# Self-identified Race and Ethnicity and How this is Perceived: Associations with the Physical and Mental Health of Incarcerated Individuals

**DOI:** 10.1007/s40615-024-02186-8

**Published:** 2024-10-04

**Authors:** Rachel A. Zajdel, Evelyn J. Patterson, Erik J. Rodriquez, Monica Webb Hooper, Eliseo J. Pérez-Stable

**Affiliations:** 1 Division of Intramural Research, National Heart, Lung, and Blood Institute (NHLBI), Bethesda, MD 20892 USA; 2 McCourt School of Public Policy, Georgetown University, Washington, DC 20057 USA; 3 Office of the Director, National Institute on Minority Health and Health Disparities (NIMHD), National Institutes of Health, 6700 Democracy Boulevard, Suite 800, Bethesda, MD 20892 USA

**Keywords:** Race and ethnicity, Perceived race, Health disparities, Incarceration

## Abstract

**Objectives:**

The singular focus on self-identified race and ethnicity in health disparities research may not fully convey the individual and structural components of experiencing race in society, or in a racialized context such as prison. Processes of racialization create boundaries between incarcerated individuals and regulate their daily interactions and access to resources, with possible effects on well-being. However, the relationship between perceived race and health has not been examined within the imprisoned population.

**Design:**

We used data from the 2016 Survey of Prison Inmates (*n* = 23,010) to assess how self-identified race, perceived race, and the discordance between racial self-identification and perception were associated with the physical (number of chronic conditions) and mental health (psychological distress) of American Indian and Alaska Native, Asian, Black, Latino, White, and multiracial incarcerated individuals.

**Results:**

Reported perception as Latino was associated with better mental and physical health relative to perception as White. Perceived Latino identity was more strongly associated with physical and mental health than a Latino self-identity. Reported perception as Black was associated with less psychological distress than perception as White, but this relationship dissipated after accounting for self-identified race. In contrast, perceived and self-identified multiracial incarcerated individuals reported worse health than their White counterparts. Having a discordant (vs. concordant) racial identity was associated with worse physical and mental health among imprisoned persons regardless of race.

**Conclusion:**

The use of a single, unidimensional measure of race and ethnicity in health disparities research does not fully reveal racialization’s influence on health, specifically for those experiencing incarceration.

**Supplementary Information:**

The online version contains supplementary material available at 10.1007/s40615-024-02186-8.

Extant knowledge regarding racial and ethnic health disparities relies on self-identified operationalizations of race and ethnicity. These measures of race and ethnicity stem from standard questions such as, “What is your race?” and “Are you of Hispanic, Latino, or Spanish origin?” However, in daily social interactions, individuals observe and interpret the race and ethnicity of others—and treat them accordingly—based on their own preconceptions in addition to others’ personal identities [1]. Consequently, sole dependence on self-identified race and ethnicity may not sufficiently reveal the individual and structural components of experiencing race and ethnicity in a racialized society [2, 3].

As such, an emerging body of literature has been challenging the singular use of self-identified race and ethnicity [3–5]. Perceived race, or the racial and ethnic categorization by others based on physical appearance, phenotypic characteristics, cultural behaviors and values, and societal conceptualizations, offers another angle to the study of racial and ethnic health disparities. Researchers measure perceived race through interviewer assignments and, most often, respondent reports regarding how they believe others usually classify their race in the US. Although this latter measurement is more akin to one’s perception of their perceived race and ethnicity, we use the term “perceived race” throughout this manuscript for simplicity.

The external ascription of race by others may be more closely linked to processes of racialization, racial discrimination, and White privilege [6], which are key mechanisms linking race to health. Perceived race may therefore affect health differently than self-identified race given that it may better capture how an individual is treated in the US’s racialized society above and beyond their personal racial self-identification. Despite increasing consideration of race as a multidimensional construct in social science research, literature on the relationship between perceived race and health remains underdeveloped. Incarcerated persons comprise a population marginalized and racialized by social institutions who have not yet been studied in the perceived race and health literature. In the present study, we use data from the 2016 Survey of Prison Inmates (SPI) to assess if self-identified race, perceived race, and perceived racial identity discordance differentially predict health outcomes among incarcerated individuals. The prison provides a unique site to further our understandings of how perceptions of race influence health outcomes in an understudied, racially stratified institution embedded in a racialized society.

## Background

### Perceived Race and Health

While research is increasingly theorizing race as multidimensional, empirical work on perceived race is still scarce relative to work on self-identified race, in part because perceived race is rarely collected in population surveys. One systematic review found only 18 articles focused on the relationship between perceived race and health, 12 of which were conducted in the US [6]. Of these studies, most (12/18) utilized a proxy measure of perceived race through respondent reports of how they believe others classify their race, while the others (6/18) assessed perceived race through interviewer assignment of race or skin tone. The results of these investigations are mixed. Several studies demonstrated a positive association between being perceived as a minoritized racial and ethnic group member and poor health-related outcomes [1, 7–10]. For example, in a nationally representative sample of respondents, Jones and colleagues found that those who self-identified as American Indian or Alaska Native (AI/AN) or Latino but reported being perceived as White were significantly more likely to report excellent or very good health relative to those whose racial self-identity of AI/AN or Latino matched how they believed they were externally ascribed [1]. Others noted similar patterns for the relationship between being perceived as a racial and ethnic minoritized individual and disadvantageous outcomes in terms of diabetes [8], receipt of preventative vaccinations [9], psychological distress [11, 12], and experiences of healthcare discrimination [9, 10].

This relationship is not consistent, though, with other studies indicating a null association between one’s perceptions of their perceived race and overall self-rated health [2, 13, 14], receipt of preventative cancer screenings [9], and self-rated mental health [14]. Additionally, one study revealed fewer depressive symptoms among respondents who self-identified as White but were interviewer-classified as Black relative to those whose racial identity as Black was concordant [4]. As these studies indicate, perceived race and health are often, but not always, associated. Multiple factors may contribute to these inconsistencies in empirical findings, including variation in methodology, health outcomes, study population, operationalization of perceived race, and the congruence between self-identified and perceived race in the target population. Further research is needed to disentangle this relationship and to address some of the weaknesses in the current literature.

There are several limitations in the existing literature on perceived race and health. First, researchers have only considered a narrow set of health outcomes [6]. The majority of studies have evaluated self-rated health [1, 2, 13, 14] or mental health [4, 11, 12, 14]. In the US, few studies have assessed the relationship between perceived race and physical health [7, 8]. Thus, while research on perceived race and health is scarce overall, the connection between perceived race and physical health is especially understudied.

Another limitation in the published literature is the focus on one or two racial and ethnic groups to the exclusion of others. Several studies appraised the relationship between perceived race and health for one self-identified racial and ethnic population, most commonly Latino individuals [2, 13, 14]. While these studies revealed noteworthy differences in health between members of the same broad racial and ethnic category, we know less about non-Latino groups. Other studies have centered on binary Black/White [7] or Latino/White [8] measures of race and ethnicity, which are limited in increasing our understanding of racialization and health in a multiracial society. Critically, the health impact of external racial ascription may differ by race and ethnicity [15], so the nuanced effects of multiple dimensions of race may be ascertained more fully with a racially heterogeneous sample.

Similarly, the congruence between self-identified and perceived race varies between racial and ethnic groups, with the greatest concordance for individuals who identify as Black or White and less concordance for individuals who identify as AI/AN, Latino, or multiracial [1, 16]. Further disparities in health may be illuminated by examining how well-being relates to the congruence between self-identified and perceived race, above and beyond the disparities revealed when assessing self-identified and perceived race individually. The present study addresses these limitations of extant research in a uniquely racialized population: incarcerated persons.

### Race, Racialization, and Incarceration

Racialization, or the institutionalization of racial categories by a social system [17], permeates the process of incarceration. Mass incarceration in the US is characterized by racial and ethnic inequality, with individuals, especially men, who identify as American Indian or Alaska Native (AI/AN), Black, and/or Latino at greatest risk of imprisonment [18, 19]. Upon incarceration, prison officials use risk-management arguments, such as minimizing the risk of violence by separating suspected gang members [20], to justify the continued racialization of imprisoned persons [21]. As racial formation theory would predict, though, the racial categories that emerge within prisons are socially constructed and highly contextual [17]. During the intake process, the prison administration, correctional officers, and incarcerated individuals themselves negotiate their racial classification from a set of prescribed categories that differ by penal facility [22]. This process results in a unique form of racial social assignment that is institutionally determined and enforced. While there is often congruence between an incarcerated individual’s racial self-identity and their institutionally determined racial classification, when disagreement occurs, it is the prison official’s categorization that prevails [22].

Individuals are compelled to embody their prescribed racial identity, regardless of alignment with their personal sense of self, due to the repercussions of violating the racial “politics” that structure daily prison life [21, 23]. For example, if an imprisoned individual requires access to a certain resource, such as a hygiene kit, they must go through their racial group’s de facto representative, who then requests the kit from a correctional officer [21]. The constant adherence to racial politics constrains freedoms and creates additional stress for imprisoned persons [24], whose individual identities are stripped and replaced by racial categories [21]. Thus, the perception of race intimately influences the everyday experiences of incarcerated individuals above and beyond their own racial self-identity. The present study explores if these unique experiences of racialization within prison are associated with the relationship between race and health in unexplored ways.

### Incarceration and Health

In addition to racialization, the criminal legal system shapes the health of over two million US residents under its domain in any given year [25]. Imprisonment can adversely impact health through various psychological, social, and physical mechanisms. Incarcerated individuals exhibit higher rates of chronic conditions [26, 27], mental illnesses [27], and infectious diseases [28] compared to the non-incarcerated population. After release, incarceration and its associated effects significantly increase the likelihood of severe health limitations [29] and depression [30] as well as reduce life expectancy [31].

Despite evidence of incarceration’s detrimental effect on health, as well as strong and consistent evidence of racial and ethnic inequalities in health in the general population [32], racial and ethnic health disparities are often reduced among imprisoned individuals [33–35]. Incarcerated individuals can be thought of as a cloistered population, in that they theoretically encounter the same physical environments, daily routines, meals, opportunities for exercise, access to health care, and limited exposure to some causes of injury and death (i.e., firearms and motor vehicles) [35]. This greater uniformity of social conditions within prisons relative to outside of prisons likely contributes to the reduction of racial and ethnic disparities in health among incarcerated individuals compared to the general population.

Moreover, the demographic composition of the prison population is not reflective of the general population in terms of race and ethnicity or socioeconomic status [19]. Similar patterns may extend to health. Since incarceration affects a higher percentage of AI/AN, Black, Latino, and multiracial individuals, it likely captures a healthier segment of these populations relative to White persons [36]. Conversely, with lower incarceration rates, imprisoned White individuals may be less healthy than the broader, generally healthier White population [36]. Therefore, any apparent reduction in racial and ethnic health disparities likely reflects social inequities in incarceration, along with social parity upon imprisonment.

### The Present Study

The present study examines if self-identified race, respondent reports of their perceived race, and the discordance between these two reports differentially predict health outcomes among the imprisoned population. To our knowledge, this is the first study to evaluate how different dimensions of race are associated with the health of incarcerated individuals, a population that is often overlooked in health disparities research [34, 37]. We aim to build upon and diversify existing knowledge on the relationship between perceived race and health in a uniquely marginalized and racialized population. We further extend upon previous research by appraising the relationship between self-identified race, perceived race, and perceived racial identity discordance and one commonly examined outcome (mental health) and one understudied outcome (physical health) given that different dimensions of race may be more strongly associated with certain health outcomes over others [38]. Lastly, we include multiple racial and ethnic groups to ascertain the nuanced effects of external racial perceptions in the US multiracial society.

This leads us to propose three hypotheses. First, studies demonstrate reduced racial and ethnic health disparities among incarcerated individuals compared to the general population [33–35]. In part, this is due to differences in health by race and ethnicity among incarcerated persons [33, 34], with incarcerated White individuals being less healthy than the general White population [36]. Furthermore, conceptualizing incarcerated individuals as a cloistered population, subject to a standardized set of living conditions, can help explain why the health of individuals within prisons may be less different than it would be outside of prison and under highly unequal social conditions [35]. Consequently, we do not expect to find perceived racial and ethnic disparities in physical health (Hypothesis 1a) or psychological distress (Hypothesis 1b).

Second, although extant findings on the relationship between perceived race and health are mixed, there are theoretical and empirical reasons to believe that perceived race more accurately captures processes of racialization in society. As racial formation theory puts forth, race is a dynamic social process [17]. Racial identities are context-specific, negotiated, and contested [17], and individuals observe and categorize an unknown person’s race and ethnicity based on their own culturally informed preconceptions. Accordingly, perceived race may encapsulate the experiences, and subsequent well-being, of incarcerated individuals better than self-identified race. Some empirical evidence supports this perspective, with studies finding that interviewer-perceived race is a stronger predictor of receiving health screenings than self-identified race [38] and respondent-perceived White social ascription can promote higher self-rated health despite a racial and ethnic minoritized self-identity [1]. We therefore hypothesize that perceived race will be a stronger predictor of physical health (Hypothesis 2a) and psychological distress (Hypothesis 2b) than self-identified race.

Third, examining perceived race in addition to self-identified race offers the opportunity to not only assess the relative strength of association of each dimension of race with health, but also to examine the relationship between having a discordant racial identity and health. A concordant racial identity results when an individual’s racial and ethnic self-identity matches how they perceive they are externally ascribed. For example, a person who identifies as Black and is perceived by others as Black has a concordant racial identity. Conversely, an individual who identifies as Black and believes they are perceived as Latino has a discordant racial identity. We expect perceived racial identity discordance to be associated with ill-health because, as affirmed by identity control theory, the dissonance between how someone identifies and how they think the world perceives them can cause distress related to the undermining of their personal identity and sense of self and can alter how they interact with others [39]. Extant findings align with the conceptualization of racial identity discordance as a stressor detrimental to well-being, with racial misclassification being linked to adverse mental and physical health outcomes [11, 40, 41]. We therefore hypothesize that having a discordant racial identity will be associated with worse physical (Hypothesis 3a) and mental health (Hypothesis 3b) compared to having a concordant racial identity.

## Data and Methods

Data analyzed came from the 2016 Survey of Prison Inmates (SPI), a nationally representative survey of persons held in state and federal prison facilities [42]. The Bureau of Justice Statistics (BJS) conducted the most recent survey from January through October 2016 through face-to-face interviews using computer-assisted personal interviewing (CAPI). The SPI gathers information regarding incarcerated individuals’ current offense, criminal history, family background and personal characteristics, and health. The response rate was 69.3% for the state prison population and 72.8% for the federal prison population [42]. We used the public use data in the present study, which is available online through the University of Michigan Inter-University Consortium for Political and Social Research (ICPSR).

### Dependent Variables

We assessed one aspect of physical health—chronic conditions—and one aspect of mental health—psychological distress. The *chronic conditions* variable was operationalized as an index count of the nine types of chronic physical illnesses contained in the SPI: hypertension, diabetes mellitus, heart conditions, cancer, stroke, arthritis, asthma, cirrhosis of the liver, and kidney conditions. Respondents who answered affirmatively to the question, “Has a doctor, nurse, or other health care provider ever told you that you had [fill in condition here]?” were coded as 1. After summing each of the conditions together, responses ranged from 0 to 9. Given that only 4.7% of respondents reported more than three conditions, we truncated the number of chronic conditions at three conditions or more.

We measured *psychological distress* using the Kessler non-specific distress scale (K6). The K6 is a six-item psychological screening instrument designed to assess the frequency of psychological distress in the past 30 days [43]. Respondents were asked how often they felt nervous, hopeless, restless or fidgety, depressed, that everything was an effort, and worthless, with answers ranging from “none of the time” (coded as 0) to “all of the time” (coded as 4). Responses were then summed to generate a score between 0 and 24. The K6 scale is internally consistent and reliable [43], across gender and education level [44] and race and ethnicity [45].

### Independent Variables

We examined the relationship between health and three dimensions of race and ethnicity: self-identified race, perceived race, and perceived racial identity discordance. We gauged *self-identified race* using responses to the question, “Which of these categories describes your race?” Respondents were able to choose from the categories American Indian or Alaska Native (AI/AN), Asian, Black or African American, Native Hawaiian or Other Pacific Islander, White, or other. A separate question asked if respondents considered themselves to be of “Spanish, Latino/Latina, or Hispanic origin.” From these questions, we created a mutually exclusive six-category race and ethnicity variable: AI/AN, Asian, Black, Latino, White, and two or more races. We excluded respondents who reported their self-identity as Native Hawaiian or Other Pacific Islander (*n* = 71) or as “other” race (*n* = 7) alone due to their small sample sizes (Table 1).

**Table 1:** Weighted descriptive statistics of 2016 Survey of Prison Inmates, by self-identified race and ethnicity (*n* = 23,010)

	AI/AN^a^	Asian	Black	Latino	White	Multiracial	Total
Demographic characteristics							
Sex (*n*, %)							
Female	113 (9.4%)	17 (3.6%)	1,107 (4.2%)	904 (5.8%)	3,007 (10.7%)	775 (9.1%)	5,923 (7.1%)
Male	218 (90.6%)	115 (96.5%)	5,910 (95.8%)	4,058 (94.2%)	5,019 (89.4%)	1,767 (90.9%)	17,087 (92.9%)
Age category (*n*, %)							
8–34 years	125 (34.5%)	51 (39.4%)	2,974 (43.4%)	2,274 (46.8%)	2,987 (36.2%)	1,003 (38.9%)	9,414 (41.3%)
35–49 years	134 (43.1%)	61 (49.7%)	2,689 (37.7%)	1,946 (38.1%)	3,093 (37.4%)	964 (37.7%)	8,887 (37.8%)
50 + years	72 (22.4%)	20 (10.9%)	1,354 (18.9%)	742 (15.1%)	1,946 (26.4%)	575 (23.4%)	4,709 (20.9%)
Education (*n*, %)							
< High school	190 (57.2%)	65 (50.7%)	4,538 (67.6%)	3,330 (69.5%)	3,805 (50.7%)	1,441 (60.2%)	13,369 (61.7%)
High school	84 (26.2%)	24 (19.4%)	1,521 (20.9%)	990 (19.9%)	2,095 (25.9%)	625 (24.1%)	5,339 (22.7%)
Some college or associates	51 (14.8%)	26 (18.0%)	728 (9.0%)	440 (7.6%)	1,470 (16.6%)	364 (11.9%)	3,079 (11.5%)
≥ College degree	6 (1.8%)	17 (11.9%)	230 (2.5%)	202 (3.0%)	656 (6.8%)	112 (3.8%)	1,223 (4.1%)
Married (*n*, %)	39 (10.6%)	27 (15.4%)	878 (12.3%)	984 (19.0%)	1,333 (15.1%)	350 (13.4%)	3,611 (14.8%)
Health insurance (*n*, %)	54 (20.1%)	47 (31.6%)	2,181 (31.0%)	1,234 (25.2%)	2,397 (31.2%)	661 (26.9%)	6,574 (29.2%)
Foreign-born (*n*, %)	3 (0.6%)	82 (59.4%)	147 (1.9%)	1,823 (33.5%)	151 (2.0%)	55 (2.4%)	2,261 (9.4%)
Language of interview (Spanish, *n*, %)	0 (0.0%)	0 (0.0%)	0 (0.0%)	1,254 (22.5%)	12 (0.2%)	2 (0.01%)	1,268 (5.1%)
Incarceration characteristics							
Crime type (*n,* %)							
Violent	179 (56.7%)	53 (46.6%)	3,711 (55.7%)	1,999 (47.9%)	3,130 (45.6%)	1,240 (54.3%)	10,312 (50.6%)
Property	49 (17.9%)	25 (18.6%)	906 (11.2%)	596 (10.9%)	1,866 (20.2)	423 (14.9%)	3,865 (14.5%)
Drug	57 (11.6%)	37 (24.4%)	1,393 (19.4%)	1,350 (23.0%)	1,659 (17.3%)	495 (16.1%)	4,991 (19.1%)
Public order	44 (13.7%)	15 (9.7%)	980 (13.4%)	978 (17.6%)	1,329 (16.5%)	368 (14.4%)	3,714 (15.4%)
Unknown	2 (0.1%)	2 (0.8%)	27 (0.3%)	39 (0.6%)	42 (0.4%)	16 (0.3%)	128 (0.4%)
Previous incarceration experiences^b^ (*n*, %)							
1 time	172 (42.1%)	102 (71.8%)	3,517 (47.7%)	2,995 (58.7%)	4,793 (55.3%)	1,409 (52.2%)	12,988 (53.1%)
2 times	63 (23.0%)	14 (13.5%)	1,644 (24.7%)	997 (20.8%)	1,582 (20.6%)	525 (23.0%)	4,825 (22.3%)
3 times	45 (15.9%)	9 (9.2%)	888 (13.2%)	506 (10.7%)	753 (11.2%)	279 (11.8%)	2,480 (11.9%)
4 times	16 (6.9%)	4 (3.4%)	447 (6.9%)	189 (4.3%)	379 (5.7%)	127 (5.5%)	1,162 (5.8%)
5 + times	32 (12.2%)	2 (2.0%)	490 (7.5%)	238 (5.5%)	444 (7.2%)	166 (7.5%)	1,372 (7.0%)
Health outcomes							
Number of chronic conditions (*n*, %)							
0 conditions	141 (45.1%)	83 (67.9%)	3,356 (49.6%)	2,881 (58.5%)	3,585 (46.5%)	1,012 (41.5%)	11,058 (49.8%)
1 condition	93 (27.3%)	24 (18.8%)	2,082 (29.3%)	1,203 (24.8%)	2,217 (27.1%)	737 (29.3%)	6,356 (27.5%)
2 conditions	47 (15.0%)	19 (10.2%)	927 (12.8%)	487 (9.6%)	1,202 (14.2%)	384 (14.4%)	3,066 (12.7%)
3 or more conditions	50 (12.6%)	6 (3.1%)	652 (8.2%)	391 (7.2%)	1,022 (12.2%)	409 (14.8%)	2,530 (9.9%)
Psychological distress (mean, sd)	7.2 (5.8)	5.6 (5.5)	6.0 (5.2)	5.6 (5.5)	6.54 (5.3)	7.3 (5.5)	6.1 (5.3)
Total (*n*, %)	331 (1.4%)	132 (0.7%)	7,017 (33.8%)	4,962 (22.3%)	8,026 (31.1%)	2,542 (10.7%)	23,010 (100%)

^a^*AI/AN* = American Indian or Alaska Native

^b^Sample size for previous incarceration experiences are AI/AN = 238, Asian = 131, Black = 6986, Latino = 4925, White = 7951, multiracial = 2506, and total = 22,827

*Perceived race* stems from the prompt: “Now I would like you to think about how other people would describe your race. Do you think they would describe you as [racial and ethnic category]?” Although this is a proxy measure of perceived race that captures one’s perceptions of how others classify them, this operationalization aligns with the most common measure of perceived race used in previous literature [6]. We created a variable with the same mutually exclusive racial and ethnic categories as above (AI/AN, Asian, Black, Latino, White, and multiracial). We excluded the small number of respondents who reported that others perceive them as Native Hawaiian or Other Pacific Islander (*n* = 40) or racially “other” (*n* = 87).

Using both measures of race, we created a *perceived racial identity discordance* variable. This measure captures if an individual’s racial self-identity maps onto their perceived race by others. Because creating a racial discordance variable for each combination of the six self-identified and perceived race categories would generate abundant classifications, including some with small sample sizes (see Table 2), we examined racial identity discordance as a binary variable (concordant = 0; discordant = 1).

**Table 2:** Self-identified race, perceived race^a^, and racial identity discordance in 2016 Survey of Prison Inmates, with weighted percent (*n* = 23,010)

		Concordant identity^b^ Perceived race						
Self-identified race		AI/AN	Asian	Black	Latino	White	Multiracial	Discordant identity^c^
AI/AN	*n*	79	0	13	10	20	209	252
	%	**23.1%**	0%	5.9%	2.8%	9.6%	58.7%	**76.9%**
Asian	*n*	0	66	5	0	1	60	66
	%	0%	**47.3%**	4.3%	0%	0.9%	47.6%	**52.7%**
Black	*n*	4	2	5,865	10	11	1,125	1,152
	%	0%	0%	**83.7%**	0.2%	0.1%	16.0%	**16.3%**
Latino	*n*	19	7	267	1,768	385	2,516	3,194
	%	0.3%	0.2%	5.9%	**37.3%**	7.2%	49.1%	**62.7%**
White	*n*	6	0	37	28	7,061	894	965
	%	0.1%	0%	0.5%	0.4%	**88.3%**	10.7%	**11.7%**
Multiracial	*n*	20	10	529	25	653	1,305	1,237
	%	0.5%	0.4%	23.5%	1.1%	25.6%	**49.0%**	**51.0%**
Column total	*n*	128	85	6,716	1,841	8,131	6,109	6,866
	%	0.5%	0.4%	32.4%	8.7%	32.0%	26.1%	**30.0%**

^a^Perceived race was measured with the question, “Now I would like you to think about how other people would describe your race. Do you think they would describe you as [racial and ethnic category]?”

^b^Sample sizes (*n*) and percentages in central column denote the proportion of the sample in each racial and ethnic subpopulation with a *concordant* self and perceived racial identity. For example, 83.7% of Black individuals in the sample have a concordant racial identity

^c^Sample sizes (*n*) and percentages in the column on the far right is the proportion of the sample in each racial and ethnic subpopulation with a *discordant* self and perceived racial identity. For example, 11.7% of White individuals have a discordant racial identity

### Covariates

We accounted for several sociodemographic variables known to shape health. *Sex* was a dichotomous variable (1 = female). *Age* was a categorical variable: 18–34 years, 35–49 years, and 50 + years. *Education* assessed respondents’ highest level of education prior to incarceration, ranging from less than high school (reference) to a college degree or more. *Married* indicated if an individual was married (1) or widowed, separated, divorced, or never married (0). *Health insurance* was a binary measure evaluating if a respondent had health insurance from any source 30 days prior to arrest (1 = yes). We also included a binary measure of birthplace (1 = foreign-born) given that it has been found to affect the relationship between perceived race and health [13]. *Language of interview* determined if the survey was conducted in English (0) or Spanish (1). Lastly, *crime type* indicated the primary category of offense a respondent was convicted of as violent (reference), property, drug, public order, or unknown.

### Analytic Strategy

Analyses comprised three parts. First, we employed zero-inflated Poisson regressions to calculate the associations between self-identified race, perceived race, and physical health. The zero-inflated Poisson best fits models with an outcome variable characterized by overdispersion and an excessive number of zeros. It is also particularly suitable in this case given that the excess zeros in the dependent variable (number of chronic conditions) may arise from different processes than the other index values (1–3 +). That is, an individual may report zero chronic conditions because they truly do not have any or because they have not received a diagnosis due to inadequate or infrequent medical care. Moreover, results from a likelihood ratio test of the equivalence of alpha to zero (*p* > 0.05) supported the choice of a zero-inflated Poisson over a zero-inflated negative binomial. We present results from the Poisson models as incidence risk ratios and results from the inflate models as coefficients, with 95% confidence intervals. An incidence risk ratio > 1.0 indicates a positive association, or an increased risk of having the health outcome in the group of interest compared to the reference group.

Second, we utilized multivariable linear regressions to similarly assess the relationships between self-identified race, perceived race, and psychological distress. For both sets of analyses (number of chronic conditions and psychological distress), the first model included only self-identified race to assess its unique influence on health, the second model included only perceived race to assess Hypothesis 1, and the third model included both self-identified race and perceived race to evaluate which dimension of race is more strongly associated with health (Hypothesis 2). In other words, the third model estimated the association of perceived race with health above and beyond the influence of self-identified race on health (and vice versa). We checked for multicollinearity in these models, given the high degree of overlap between self-identified and perceived race for some of the racial and ethnic groups. The mean variance inflation factor for the models, using binarily coded versions of the categorical variables, was 1.81. All variance inflation factors were below the standard threshold of 5 [46], with the exception of the binary variables for Black self-identified and perceived race (5.11 and 5.04, respectively). Since multicollinearity can inflate standard errors, the statistical significance of the coefficients for Black self-identified and perceived race may be underestimated.

Third, we used zero-inflated Poisson regression and linear regression to generate associations between perceived racial identity discordance and number of chronic conditions or psychological distress, respectively, to test Hypothesis 3. We then explored these findings as predicted probabilities using linear regression. Predicted probabilities reveal the average number of chronic conditions and level of psychological distress for individuals with a perceived concordant vs. discordant racial identity when covariates are kept at the mean. For the predicted probability estimates, we modeled chronic conditions as the untruncated number of conditions. All regression models controlled for sex, age, education, marital status, health insurance, birthplace, language of interview, and crime type.

Our analytic sample included respondents without missing information for any of the variables of interest. This resulted in a sample of 23,010, representing 92.6% of all individuals surveyed. We utilized analysis weights and replicate weights using the *svyset* command and jackknife method of variance estimation, as outlined in the SPI User Guide [47]. This accounts for survey design, nonresponse, and post-stratification adjustment and thereby produces national estimates of the US prison population. Analyses were conducted in Stata-17.

## Results

Table 1 presents descriptive statistics by race and ethnicity and overall. The most frequently reported self-identified race was Black (33.8%), followed by White (31.1%), Latino (22.3%), multiracial (10.7%), AI/AN (1.4%), and Asian (0.7%). A minority of respondents were female (7.1%), married (14.8%), foreign-born (9.4%), conducted the interview in Spanish (5.1%), or had health insurance prior to incarceration (29.2%). Respondents tended to be younger (41.3% below 35 years old) and have less than a high school education (61.7%). Half of respondents were convicted of a violent crime type (50.6%). Over half of respondents were currently serving their first prison term (53.1%). Nearly 50% of incarcerated individuals reported no physical health conditions and over a quarter reported one diagnosed illness. Respondents reported an average of 6.1 on the psychological distress scale. Notably, the distribution of these social and health characteristics tended to vary by race and ethnicity.

Table 2 breaks down racial identity, perceived race, and the concordance/discordance of these two variables. The most commonly reported perceived races were Black (32.4%) and White (32.0%). Most respondents reported a perceived concordant racial identity (70.0%), although 30.0% reported a perceived discordant racial identity. Racial identity concordance was highest for self-identified White (88.3%) and Black (83.7%) respondents but much lower for self-identified multiracial (49.0%), Asian (47.3%), Latino (37.3%), and AI/AN (23.1%) respondents.

### Self-Identified Race, Perceived Race, and Physical Health

Table 3 presents results demonstrating the relationships between self-identified race, perceived race, and health. All models included sociodemographic covariates, which were associated with health in the expected directions, with few exceptions (e.g., higher levels of education were not protective; see Online Resource 1). Self-identification as Asian (incidence risk ratio (IRR), 0.69; C.I., 0.52, 0.90; *p* < 0.01) or Latino (IRR, 0.91; C.I., 0.86, 0.97; *p* < 0.01) was inversely associated with the number of chronic conditions relative to self-identification as White. In contrast, self-identification as multiracial was positively associated with the number of chronic conditions relative to self-identification as White (IRR, 1.15; C.I., 1.09, 1.21; *p* < 0.001).

**Table 3:** Self-identified race, perceived race, and health among incarcerated individuals (*n* = 23,010)

	Model 1: Self-identified race		Model 2: Perceived race^b^		Model 3: Self-identified and perceived race	
Number of chronic conditions^a^	Incidence risk ratio	[CI]	Incidence risk ratio	[CI]	Incidence risk ratio	[CI]
Self-identified race (ref = White)						
AI/AN	1.06	[0.91–1.24]	-	-	1.07	[0.89–1.28]
Asian	0.69**	[0.52–0.90]			0.70*	[0.52–0.96]
Black	0.96	[0.92–1.00]	-	-	0.91*	[0.84–0.99]
Latino	0.91**	[0.86–0.97]	-	-	0.93	[0.86–1.00]
Multiracial	1.15***	[1.09–1.21]			1.11**	[1.04–1.18]
Perceived race (ref = White)						
AI/AN	-	-	0.85	[0.68–1.05]	0.82	[0.64–1.05]
Asian			0.69	[0.47–1.01]	0.87	[0.57–1.31]
Black	-	-	0.98	[0.94–1.02]	1.06	[0.98–1.16]
Latino	-	-	0.78***	[0.71–0.86]	0.83**	[0.74–0.93]
Multiracial			1.05*	[1.00–1.10]	1.08*	[1.01–1.14]
Psychological distress^c^	Coefficient	[CI]	Coefficient	[CI]	Coefficient	[CI]
Self-identified race (ref = White)						
AI/AN	0.60	[–0.20–1.40]	-	-	0.79	[− 0.19–1.78]
Asian	− 0.38	[–1.45–0.69]			− 0.37	[− 1.62–0.87]
Black	− 0.54***	[–0.77– − 0.30]	-	-	− 0.18	[− 0.61–0.25]
Latino	− 0.38	[–1.45–0.69]	-	-	− 0.27	[− 0.70–0.17]
Multiracial	0.62***	[0.33–0.91]			0.74***	[0.37–1.11]
Perceived race (ref = White)						
AI/AN	-	-	− 0.11	[− 1.31–1.10]	− 0.60	− 2.07–0.88]
Asian			− 0.28	[− 1.70–1.14]	− 0.06	[− 1.68–1.56]
Black	-	-	− 0.59***	[− 0.84– –0.35]	− 0.44	[− 0.88–0.01]
Latino	-	-	− 1.22***	[− 1.66– − 0.78]	− 1.01***	[− 1.50– − 0.53]
Multiracial			− 0.02	[− 0.26–0.21]	− 0.02	[− 0.35–0.31]

Authors’ calculations using data from the 2016 Survey of Prison Inmates. Weighted statistics from zero-inflated Poisson regression for number of chronic conditions model and linear regression for psychological distress model

*p < .05; **p < .01; ***p < .001

All models include sex, age, education, marital status, health insurance status, foreign-born status, language of interview, and crime type. Full results available in Appendix Tables 1-2

^a^Number of chronic conditions is an index count ranging from 0 to 3 + that includes diabetes mellitus, hypertension, heart conditions, arthritis, asthma, cancer, stroke, liver cirrhosis, and kidney conditions

^b^Perceived race was measured with the question, “Now I would like you to think about how other people would describe your race. Do you think they would describe you as [racial and ethnic category]?”

^c^Psychological distress is measured with the Kessler (K6) non-specific distress scale

Similarly, reporting a perceived Latino identity was inversely associated with the number of chronic conditions (IRR, 0.78; C.I., 0.71, 0.86; *p* < 0.001), while reported perception as multiracial was positively associated with the number of chronic conditions (IRR, 1.05; C.I., 1.00, 1.10; *p* < 0.05). Incarcerated persons reportedly perceived as Black, AI/AN, or Asian exhibited a similar number of chronic conditions to those reportedly perceived as White. These results provide partial support for Hypothesis 1a.

Findings were generally robust to the addition of both measures of race in the same model (Table 3, Model 3). That is, self-identification as Asian (IRR, 0.70; C.I., 0.52, 0.96; *p* < 0.05) continued to be inversely related to the number of chronic conditions, while self-identification as multiracial (IRR, 1.11; C.I., 1.04, 1.18; *p* < 0.01) continued to be positively related to the number of chronic conditions relative to self-identification as White. Self-identified Latino persons no longer had a significantly reduced risk of reporting a higher number of chronic conditions upon controlling for perceived race, though. In addition, self-identification as Black became significantly associated with physical health upon controlling for perceived race (IRR, 0.91; C.I., 0.84, 0.99; *p* < 0.05). Reporting being perceived as Latino remained significantly and inversely associated with the number of chronic conditions (IRR, 0.83; C.I., 0.74, 0.93; *p* < 0.01) and reporting being perceived as multiracial continued to be positively associated with the number of chronic conditions (IRR, 1.08; C.I., 1.01, 1.14; *p* < 0.05) relative to reporting being perceived as White. Hypothesis 2a is thus partially supported.

### Self-Identified Race, Perceived Race, and Psychological Distress

Results for the relationship between self-identified race, perceived race, and psychological distress are also presented in Table 3. Coefficients for the sociodemographic covariates are shown in Online Resource 2 and generally follow expected patterns. Self-identification as Black was associated with lower levels of psychological distress (*β*, − 0.54; C.I., − 0.77, − 0.30; *p* < 0.001), while self-identification as multiracial was associated with heightened levels of psychological distress (*β*, 0.62; C.I., 0.33, 0.91; *p* < 0.001).

Reporting being perceived as Black (*β*, − 0.59; C.I., − 0.84, − 0.35; *p* < 0.001) or Latino (*β*, − 1.22; C.I., − 1.66, − 0.78; *p* < 0.01) was associated with lower psychological distress scores, but reporting being perceived as AI/AN, Asian, or multiracial was not significantly associated with psychological distress. These latter findings support Hypothesis 1b.

When both measures of race were included in the model (Table 3, Model 3), neither self-identification nor perception as Black were associated with psychological distress. However, self-identification as multiracial was still associated with higher psychological distress scores (*β*, 0.74; C.I., 0.37, 1.11; *p* < 0.001) and reporting being perceived as Latino remained significantly associated with lower levels of psychological distress (*β*, − 1.01; C.I., − 1.50, − 0.53; *p* < 0.001). Results provide limited supported for Hypothesis 2b.

### Perceived Racial Identity Discordance and Health

**Fig. 1:**
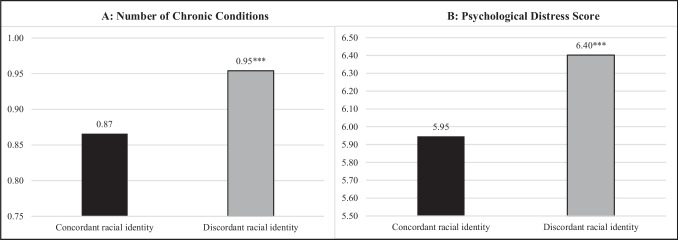
Perceived racial identity discordance and predicted probability of the number of chronic conditions^a^ (**A**) and psychological distress^b^ (**B**) score among incarcerated individuals (*n* = 23,010). Notes: Authors’ calculations using data from the 2016 Survey of Prison Inmates. Weighted statistics from linear regressions. Model controls for sex, age, education, birthplace, language of interview, marital status, health insurance, and crime type. *p < .05; **p <.01; ***p <.001 indicates difference from a perceived concordant racial identity. ^a^ Number of chronic condition includes the following: diabetes mellitus, hypertension, heart conditions, arthritis, asthma, cancer, stroke, liver cirrhosis, or kidney conditions. ^b^ Psychological distress is measured with the Kessler (K6) non-specific distress scale

Table 4 presents findings for the relationship between perceived racial identity discordance and health. As predicted in Hypothesis 3a and 3b, compared to having a perceived concordant racial identity, having a perceived discordant racial identity was positively associated with the number of chronic conditions (IRR**,** 1.09; C.I., 1.05, 1.14; *p* < 0.001) and psychological distress (*β*, 0.46; C.I., 0.28, 0.63; *p* < 0.001). Figure 1 further depicts these patterns. Respondents with a perceived discordant racial identity had an average of 0.95 chronic conditions compared to 0.87 conditions among those with a concordant racial identity (*p* < 0.001). Moreover, respondents with a discordant racial identity reported an average of 6.40 symptoms of psychological distress compared to 5.95 symptoms among those with a concordant racial identity (*p* < 0.001).

**Table 4:** Perceived racial identity discordance, number of chronic conditions^a^, and psychological distress^b^ among incarcerated individuals (*n* = 23,010)

	Panel A: Number of chronic conditions		Panel B: Psychological distress	
	Incidence risk ratio	[CI]	Coefficient	[CI]
Perceived racial identity discordance	1.09***	[1.05–1.14]	0.46***	[0.28–0.63]
Female	1.34***	[1.28–1.40]	1.45***	[1.14–1.77]
Age (ref = 18–34 years)				
35–49 years	1.72***	[1.63–1.82]	− 0.09	[− 0.29–0.12]
50 + years	3.15***	[2.98–3.34]	− 0.49**	[− 0.77– –0.20]
Education (ref = < HS)				
High school	0.96	[0.92–1.01]	− 0.47***	[− 0.66– –0.28]
Some college	1.05*	[1.00–1.10]	− 0.42**	[− 0.68– –0.15]
College degree or higher	1.05	[0.98–1.13]	− 0.45	[− 0.91–0.02]
Married	1.12***	[1.07–1.17]	0.01	[− 0.23–0.26]
Health Insurance	0.98	[0.94–1.02]	− 0.88***	[− 1.05– –0.71]
Foreign-born	0.78***	[0.70–0.88]	− 1.02***	[− 1.46– − 0.58]
Language of interview	0.78**	[0.68–0.90]	− 0.80**	[− 1.34– − 0.25]
Crime type (ref = violent)				
Property	0.94*	[0.89–0.99]	− 0.32*	[− 0.58– − 0.06]
Drug	0.87***	[0.83–0.92]	− 1.13***	[− 1.44– − 0.83]
Public order	0.93*	[0.88–0.99]	− 0.68***	[− 0.93– − 0.42]
Unknown	1.02	[0.80–1.31]	− 0.49	[− 1.77–0.79]
Constant	0.53***	[0.50–0.56]	6.91***	[6.63–7.18]
Inflate model constant^c^	− 2.36***	[− 2.57– − 2.14]	-	-

Authors’ calculations using data from the 2016 Survey of Prison Inmates. Weighted statistics from zero-inflated Poisson regression in panel A and linear regression in panel B

*p < .05; **p < .01; ***p < .001

^a^Number of chronic conditions is an index count ranging from 0 to 3 + that includes diabetes mellitus, hypertension, heart conditions, arthritis, asthma, cancer, stroke, liver cirrhosis, and kidney conditions

^b^Psychological distress is measured with the Kessler (K6) non-specific distress scale

^c^Results from the inflate model are presented as coefficients

## Discussion and Conclusion

The present study assessed how different dimensions of race distinctively predict physical and mental health among incarcerated individuals. Evidence of parity in physical and mental health for some perceived racial and ethnic groups does not completely align with health patterns in the general population by self-identified race and ethnicity. For example, AI/AN and Black individuals tend to exhibit higher rates of physical morbidity and multiracial individuals tend to experience higher levels of psychological distress relative to White individuals [32, 48, 49], but results based on our proxy measure of perceived race did not parallel these trends. Instead, these findings aligned with previous research demonstrating that the racial and ethnic health disparities observed within the prison population can differ from those observed in the general population [33–35]. The present study extends upon this work to show that unique racial and ethnic health disparities within the incarcerated population can also be seen when investigating perceived race.

Divergent racial and ethnic disparities in physical health conditions and functioning in prison may be produced by differential health by race and ethnicity among those who are selected into prison [33, 34] as well as the increased uniformity of social conditions by race and ethnicity within prison walls relative to the outside [35]. That is, US society is highly stratified, with institutional racism, residential segregation, insufficient social services, access to healthcare, and other structural forces generating racial and ethnic health disparities. While imprisoned, though, individuals are theoretically subjected to the same living conditions and have access to the same resources, including healthcare [35]. This uniformity of conditions creates a cloistered population and may counter previously experienced social deprivation to lessen health inequalities by perceived race and ethnicity within prisons. Importantly, though, stark disparities in health remained for individuals who believed they were perceived as multiracial in terms of physical health, and for self-identified multiracial individuals in terms of both physical and mental health. Thus, not all racial and ethnic groups or health outcomes benefitted from greater social parity in prison. More broadly, this finding suggests that racialization and related processes that occur both outside and inside prison affect the health of incarcerated individuals and points to a need for increased consideration of multiracial individuals in health disparities research.

Findings also support the notion that perceived race may be capturing different aspects of the racialized experiences of incarcerated individuals compared to self-identified race. Whether perceived race was a stronger predictor of health than self-identified race depended on the specific racial categorization. Compared to perceived race, self-identified race was a stronger predictor of physical health for Black, Asian, and multiracial respondents and psychological distress for multiracial respondents. Although these specific findings were contrary to expectations, the absent or weakened association between perceived race and health for these racial and ethnic groups is consistent with much of the existing literature [6]. Further research is needed to disentangle the mechanisms linking perceived race to health.

In contrast, although self-identification as Latino trended in the same direction, reporting being perceived as Latino was more strongly associated with physical and mental health than self-identification as Latino. Interestingly, the Latino population comprised the racial and ethnic group in this study with the second highest level of perceived racial identity discordance. Only 37.3% of self-identified Latino individuals reported that they believe others perceive them as Latino alone. Therefore, incarcerated Latino individuals are consistently racialized as something other than Latino—and likely treated accordingly. Future research should explore the health implications of having one’s racial self-identity so commonly misclassified, as is the case for incarcerated Latino individuals, given that the US racial hierarchy confers privilege to individuals perceived as White, and, conversely, disadvantages to members of historically oppressed racial and ethnic groups [6, 16].

Lastly, compared to individuals with a concordant racial identity, those with a discordant racial identity were more likely to have a higher number of chronic conditions and a higher level of psychological distress. Individuals with a discordant racial identity may experience strain related to their personal identity, sense of self, and experiences in interactions with others [11, 16, 39, 50], as many multiracial individuals report [51]. Perceived autonomy in choosing one’s racial identity, discordance between one’s racial self-identity and external ascription, and sense of belonging are in turn associated with elevated depressive symptoms and stress [52]. Future research should disentangle how different patterns of racial identity discordance (e.g., self-identified White, perceived as Black) are related to health in the context of the US’s system of racial stratification and White privilege.

The present study has some limitations. First, the sample sizes for individuals who self-identified or were reportedly perceived as AI/AN or Asian were relatively small, so results for these populations should be interpreted with caution. In addition, this research relied on self-reported measures of chronic physical health conditions. Given that incarcerated individuals comprise a particularly marginalized social group, many imprisoned persons do not have access to healthcare in their home communities and therefore may not have been diagnosed with a condition. Nevertheless, the legislatively mandated provision of healthcare within prisons alleviates some of this concern. A similar limitation arises with the measurement of perceived race, which relies on respondents’ perceptions of how others view their race, which may or may not align with how they are commonly perceived. Additionally, factors such as prior health and length of incarceration may have had an influence on the health status of respondents, but this information was (a) tied up with questions about current health and (b) suppressed in the dataset, respectively. Lastly, it was beyond the scope of the present study to test the mechanisms through which perceived race operates to shape health. Future research should test the roles of discrimination, bias, and stress due to racial identity discordance as possible pathways that link perceived race with health.

Despite these limitations, this was the first study to evaluate how multiple dimensions of race predicted the physical and mental health of incarcerated individuals. The process of racialization influences how individuals are viewed and treated by others and affects their chances of encountering institutions such as the criminal legal system. Upon incarceration, an individual’s personal identity is stripped and replaced by an institutionally determined racial category [21]. Incarcerated persons’ daily lives, and access to resources, are constrained by this racial classification, whether it aligns with their self-identification or not [21, 24]. Thus, we examined if perceived race was differentially associated with health compared to self-identified race since it may more accurately capture the experiences of incarcerated individuals in this uniquely racialized setting. Results from the present study suggest that the use of a single, unidimensional measure of race in health disparities research may not reflect the full extent, or nuanced patterns, of the relationship between racialization and health for incarcerated individuals. Perceived race may therefore provide utility in capturing the experiences of some individuals above and beyond self-reported race.

## Supplementary Information

Below is the link to the electronic supplementary material.

Supplementary file1 (DOCX 24 KB)

## Data Availability

Data is distributed online through the University of Michigan Inter-University Consortium for Political and Social Research (ICPSR).

## Code Availability

Stata code available upon request.
