# Supplementary material for: Self-identified Race and Ethnicity and How this is Perceived: Associations with the Physical and Mental Health of Incarcerated Individuals
Source: J Racial Ethn Health Disparities. Author manuscript; Available in PMC 2025 Nov 25. (PMC12188577; doi:10.1007/s40615-024-02186-8)
Supplement: Supplementary file 1 [file NIHMS2088943-supplement-Supplemental_Tables.docx]

| Supplemental Table 1. Self-Identified Race, Perceived Race^a^, and Number of Chronic Conditions^b^ among Incarcerated Individuals (n=23,010) |  |  |  |  |  |  |
| --- | --- | --- | --- | --- | --- | --- |
|  | Model 1: Self-identified race |  | Model 2: Perceived race |  | Model 3: Self-identified and perceived race |  |
|  | Incidence risk ratio | [CI] | Incidence risk ratio | [CI] | Incidence risk ratio | [CI] |
| Self-identified race (ref=White) |  |  |  |  |  |  |
| AI/AN | 1.06 | [0.91-1.24] | - | - | 1.07 | [0.89-1.28] |
| Asian | 0.69** | [0.52-0.90] |  |  | 0.70* | [0.52-0.96] |
| Black | 0.96 | [0.92-1.00] | - | - | 0.91* | [0.84-0.99] |
| Latino | 0.91** | [0.86-0.97] | - | - | 0.93 | [0.86-1.00] |
| Multiracial | 1.15*** | [1.09-1.21] |  |  | 1.11** | [1.04-1.18] |
| Perceived race (ref=White) |  |  |  |  |  |  |
| AI/AN | - | - | 0.85 | [0.68-1.05] | 0.82 | [0.64-1.05] |
| Asian |  |  | 0.69 | [0.47-1.01] | 0.87 | [0.57-1.31] |
| Black | - | - | 0.98 | [0.94-1.02] | 1.06 | [0.98-1.16] |
| Latino | - | - | 0.78*** | [0.71-0.86] | 0.83** | [0.74-0.93] |
| Multiracial |  |  | 1.05* | [1.00-1.10] | 1.08* | [1.01-1.14] |
| Female | 1.32*** | [1.26-1.38] | 1.33*** | [1.27-1.39] | 1.32*** | [1.26-1.38] |
| Age (ref=18-34 years) |  |  |  |  |  |  |
| 35-49 years | 1.71*** | [1.62-1.80] | 1.72*** | [1.63-1.81] | 1.71*** | [1.62-1.81] |
| 50+ years | 3.09*** | [2.93-3.27] | 3.12*** | [2.95-3.30] | 3.10*** | [2.93-3.28] |
| Education (ref=<HS) |  |  |  |  |  |  |
| High school | 0.95* | [0.91-1.00] | 0.96 | [0.92-1.00] | 0.96 | [0.91-1.00] |
| Some college | 1.04 | [0.99-1.09] | 1.04 | [0.99-1.09] | 1.04 | [0.99-1.09] |
| College degree or higher | 1.04 | [0.97-1.12] | 1.04 | [0.97-1.12] | 1.04 | [0.97-1.12] |
| Married | 1.12*** | [1.07-1.18] | 1.12*** | [1.07-1.17] | 1.12*** | [1.08-1.18] |
| Health Insurance | 0.98 | [0.94-1.02] | 0.97 | [0.94-1.01] | 0.98 | [0.94-1.01] |
| Foreign-born | 0.84** | [0.75-0.95] | 0.83** | [0.74-0.94] | 0.86* | [0.76-0.97] |
| Language of interview | 0.81** | [0.70-0.93] | 0.82** | [0.71-0.95] | 0.83* | [0.71-0.96] |
| Crime type (ref=violent) |  |  |  |  |  |  |
| Property | 0.94* | [0.89-0.99] | 0.94* | [0.89-0.99] | 0.94* | [0.89-0.99] |
| Drug | 0.87*** | [0.83-0.92] | 0.87*** | [0.83-0.91] | 0.87*** | [0.83-0.92] |
| Public order | 0.93* | [0.88-0.99] | 0.93* | [0.88-0.99] | 0.93* | [0.88-0.99] |
| Unknown | 1.03 | [0.80-1.32] | 1.02 | [0.80-1.31] | 1.03 | [0.80-1.32] |
| Constant | 0.56*** | [0.52-0.59] | 0.55*** | [0.52-0.59] | 0.55*** | [0.51-0.59] |
| Inflate model constant^c^ | -2.38*** | [-2.60--2.16] | -2.37*** | [-2.59--2.15] | -2.41*** | [2.63--2.18] |
| Notes: Author’s calculations using data from the 2016 Survey of Prison Inmates. Weighted statistics from zero-inflated Poisson regression. *p < .05; **p <.01; ***p <.001. ^a^ Perceived race was measured with the question, “Now I would like you to think about how other people would describe your race. Do you think they would describe you as [racial and ethnic category]?” ^b ^Number of chronic conditions is an index count ranging from 0-3+ that includes diabetes mellitus, hypertension, heart conditions, arthritis, asthma, cancer, stroke, liver cirrhosis, and kidney conditions. ^c^ Results from the inflate model are presented as coefficients. |  |  |  |  |  |  |

| Supplemental Table 2. Self-Identified Race, Perceived Race^a^, and Psychological Distress^b^ among Incarcerated Individuals (n=23,010) |  |  |  |  |  |  |  |
| --- | --- | --- | --- | --- | --- | --- | --- |
|  | Model 1: Self-identified race |  | Model 2: Perceived race |  |  | Model 3: Self-identified and perceived race |  |
|  | Coefficient | [CI] |  | Coefficient | [CI] | Coefficient | [CI] |
| Self-identified race (ref=White) |  |  |  |  |  |  |  |
| AI/AN | 0.60 | [-0.20-1.40] |  | - | - | 0.79 | [-0.19-1.78] |
| Asian | -0.38 | [-1.45-0.69] |  |  |  | -0.37 | [-1.62-0.87] |
| Black | -0.54*** | [-0.77--0.30] |  | - | - | -0.18 | [-0.61-0.25] |
| Latino | -0.38 | [-1.45-0.69] |  | - | - | -0.27 | [-0.70-0.17] |
| Multiracial | 0.62*** | [0.33-0.91] |  |  |  | 0.74*** | [0.37-1.11] |
| Perceived race (ref=White) |  |  |  |  |  |  |  |
| AI/AN | - | - |  | -0.11 | [-1.31-1.10] | -0.60 | [-2.07-0.88] |
| Asian |  |  |  | -0.28 | [-1.70-1.14] | -0.06 | [-1.68-1.56] |
| Black | - | - |  | -0.59*** | [-0.84--0.35] | -0.44 | [-0.88-0.01] |
| Latino | - | - |  | -1.22*** | [-1.66--0.78] | -1.01*** | [-1.50--0.53] |
| Multiracial |  |  |  | -0.02 | [-0.26-0.21] | -0.02 | [-0.35-0.31] |
| Female | 1.35*** | [1.04-1.67] |  | 1.36*** | [1.05-1.68] | 1.33*** | [1.02-1.65] |
| Age (ref=18-34 years) |  |  |  |  |  |  |  |
| 35-49 years | -0.13 | [-0.34-0.07] |  | -0.11 | [-0.31-0.10] | -0.13 | [-0.33-0.07] |
| 50+ years | -0.61*** | [-0.89--0.33] |  | -0.58*** | [-0.86--0.29] | -0.62*** | [-0.90--0.34] |
| Education (ref=<HS) |  |  |  |  |  |  |  |
| High school | -0.55*** | [-0.74--0.35] |  | -0.54*** | [-0.73--0.34] | -0.55*** | [-0.75--0.36] |
| Some college | -0.53*** | [-0.80--0.26] |  | -0.53*** | [-0.80--0.26] | -0.55*** | [-0.82--0.28] |
| College degree or higher | -0.57* | [-1.04--0.10] |  | -0.60* | [-1.07--0.13] | -0.60* | [-1.07--0.14] |
| Married | 0.03 | [-0.21-0.28] |  | 0.02 | [-0.22-0.27] | 0.03 | [-0.21-0.28] |
| Health Insurance | -0.86*** | [-1.03--0.68] |  | -0.88*** | [-1.06--0.71] | -0.87*** | [-1.04--0.69] |
| Foreign-born | -0.74** | [-1.19--0.28] |  | -0.79*** | [-1.23--0.35] | -0.67** | [-1.12--0.22] |
| Language of interview (ref=English) | -0.58* | [-1.15--0.00] |  | -0.57 | [-1.15-0.01] | -0.49 | [-1.07-0.10] |
| Crime type (ref=violent) |  |  |  |  |  |  |  |
| Property | -0.40** | [-0.65--0.14] |  | -0.40** | [-0.65--0.14] | -0.40** | [-0.65--0.14] |
| Drug | -1.13*** | [-1.44--0.81] |  | -1.14*** | [-1.46--0.83] | -1.13*** | [-1.44--0.81] |
| Public order | -0.70*** | [-0.96--0.45] |  | -0.70*** | [-0.95--0.45] | -0.69*** | [-0.95--0.44] |
| Unknown | -0.46 | [-1.73-0.82] |  | -0.51 | [-1.79-0.77] | -0.47 | [-1.75-0.80] |
| Constant | 7.35*** | [7.03-7.66] |  | 7.40*** | [7.09-7.72] | 7.37*** | [7.05-7.68] |
| Notes: Author’s calculations using data from the 2016 Survey of Prison Inmates. Weighted statistics from logistic regression. *p < .05; **p <.01; ***p <.001. ^a^ Perceived race was measured with the question, “Now I would like you to think about how other people would describe your race. Do you think they would describe you as [racial and ethnic category]?” ^b ^Psychological distress is measured with the Kessler K6 non-specific distress scale. |  |  |  |  |  |  |  |
